# Current News and Opinion

**Published:** 1886-11

**Authors:** 


### Current News and Opinion.

### DEVIATION OF THE NASAL SEPTUM.

Although the introduction of the laryngoscope, nearly thirty years ago, rapidly developed a new era in the diagnosis and treatment of diseases of the larynx, it is a much shorter time since greater attention has been paid by specialists to affections of the nose and its adjacent parts. In the July number of *The American Journal of the Medical Sciences*, Dr. J. W. Gleitsmann, of New York, in an instructive paper on deviation of the nasal septum, points out the different important functions performed by the nose in the human economy, and the results of interference with these functions. The upper part of the nasal cavity, the olfactory region, presides over the sense of smell, whilst the lower one, the respiratory region, is the normal way for the air during the act of respiration. Interference with this natural channel leads to mouth-breathing with its manifold subsequent evils. When the air passes through the nose, it is not only cleansed and moistened, but it also reaches the lungs much warmer than when breathing is going on by the mouth. Nasal respiration with closed lips further exerts a negative pressure of two to four mercury milligrammes in the oral cavity, by which the tongue is drawn to the hard palate, and the muscular action, maintaining the position of the lower maxilla, greatly assisted. The nose also acts the part of a resonant chamber for the human voice, and nasal obstruction imparts to it the so-called dead character, described in Meyer's paper on adenoid vegetations. Finally, it is due to the anatomical relations of the nose to the eye and ear that cases of catarrhal conjunctivitis and lachrymal fistula frequently heal only when co-existing nasal affections are relieved, and that the latter are, in an overwhelming number of instances, often productive of aural diseases of the severest kind. Aside from the symptoms of nasal stenosis in a greater or less degree, deviations of the septum, Dr. Gleitsmann points out, are apt to cause disfigurement of the face, and also have some relation to the bony structures of the head, which he fully explains. The pathology, etiology, symptomatology and treatment of these deviations is fully discussed.

—*Exchange.*

---

### FROM BEN JONSON, (1609.)

A lady should, indeed, study her face when we think she sleeps; nor, when the doors are shut, should men be inquiring; all is sacred within, then. Is it for us to see their perukes put on, *their false teeth*, their complexion, their eyebrows, their nails? You see gilders will not work but enclosed. They must not discover how little serves, with the help of art, to adorn a great deal.

*The Silent Woman.* Act I, Scene 1.

A most vile face, and yet she spends me forty pound a year in mercury and hog's bones. All *her teeth* were made in the Black-friars, both her eyebrows in the Strand, and her hair in Silver Street.

*The Same.* Act IV, Scene 1.

### A PROBLEM IN BACTERIOLOGY.

A curious feature in the life-history of disease germs is the sudden virulence they frequently acquire on being communicated to men and animals of differing environments. Bates, in his reports of travels in the upper valley of the Amazon, gives an account of a tribe of Indians, who, though ordinarily free from consumption, suffer terribly from the disease on coming in contact with the whites, these latter being hardy travellers and also free from the disease. The history of Texas or Spanish cattle fever is generally known. Of the cattle raised in Texas, none are ever seen to suffer from this fever. If a cow is taken there the probability is she will die by the time she has her first calf. If the calf is not still-born it is apt to be free from the fever. When Texas cattle are driven north, they communicate the disease very readily to native cattle, though, as has been already said, they may not have the disease themselves, and may seem never to have had it.

But perhaps the most curious manifestations of the influence of disease germs yet mentioned is the communication of "stranger's cold" to the natives of St Kilda, a small island north of Ireland. The inhabitants, who number about seventy-five or eighty, declare that they invariably have epidemics of "colds" when they are visited by strangers, which happens two or three times a year. The island of St. Kilda embraces less than five square miles and the inhabitants have married in-and-in for perhaps one thousand years, never having numbered more than three hundred souls at any one time After this long history of intermarriages, they are said to be singularly free from mental and physical defects, the only peculiarity exhibited being this susceptibility to cold, as above mentioned, and a tendency on the part of the children to die of trismus nascentium, full fifty per cent. of the new-born dying from the latter cause.

These curious facts form a portion of the store of knowledge which must be gathered and collated before philosophers can give us a comprehensive and satisfactory system of bacteriology.—*American Practitioner and News.*

---

### MASSACHUSETTS DENTAL SOCIETY.

The twenty-second annual meeting of the Massachusetts Dental Society will be held in the Y. M. C. A. building, corner Berkeley and Boylston Streets, Boston, on Thursday and Friday, December 9 and 10; 1886, commencing at 11 o'clock Thursday morning.

A collation will be served at the close of the afternoon session on Thursday, to which ladies are invited, and the evening session will be held in the dining-hall. Tickets for collation will be 75 cents each, and members are requested to notify the Secretary of the number they desire.

Programmes of the meeting will be sent later. Members are requested to give immediate notice of any change of address, that the copy may be prepared for the printer.

G. F. Eames, Sec'y, pro tem

62 Trinity Terrace, Boston.

### COCAINE AIDED BY ELECTRICITY TO ACT AS LOCAL ANÆSTHETIC.

O ! thou glorious drug; what virtues dost thou not possess ! Truthfully, after a time it will be easier to enumerate the virtues wanting to, than those possessed by cocaine. It is well known that the simple application of a cocaine solution upon the sound skin has no anæsthetic effect. This may, however, be brought about, according to Dr. Wagner, of Vienna, by combining an electric current with the cocaine. It is known that the electric current has the property of causing the forward movements of fluids which are contained in capillary tubes—this is called the cataphoric force or effect of the electric current. If the electrodes of a battery are moistened with a solution of cocaine, it has been found that the latter is propelled into the cellular tissue, causing the skin to become anæsthetic within a few minutes, so that it may be punctured with needles or cut with knives, without causing any sensation or feeling of pain. Any desired amount of skin surface may in this manner be made anæsthetic. The anæsthesia lasts ten to fifteen minutes, but may be prolonged by applying an Esmarch bandage previous to the application of cocaine. This is a most frequent suggestion, and we would be glad to publish any experiences that our readers may have in this line.—*The Medical and Surgical Reporter.*

---

### THE NATURE OF THE PERICEMENTAL MEMBRANE.

In an article on "Exostosis," read before the Connecticut Valley Dental Society and published in the August number of this journal, we inadvertently misstated the theory of the nature of the peridental membrane as held by Dr. L. C. Ingersoll, transposing his views and those of the English school of fifty years ago. Dr. Ingersoll's position is that the membrane is dual, but that the layer on the cemental side is essentially different from that on the osseous side—that the two "layers," "parts," "varieties," have nearly the same origin—that one originates in the osseous system, and the other in the dental system, within the dental follicle, in the family of the dental tissues, and that in no sense is one part a doubling of the other part, or a reflection of either part back upon itself—that the duality is entirely of another sort—that while they are different in many other respects, they are chiefly different in their structures and in their *origin*—that they come from opposite directions and unite in the socket—that they are cotemporaneous in their development from the necessity of a cotemporaneous development of tooth-socket and tooth-root.

---

According to the Statistical Report of the Registrar-General of Great Britain, death plays greater havoc in the ranks of physicians than in those of any other profession. Here are the figures, giving the death rate per 1000 :

| | | | |
|---|---|---|---|
| Clergymen | 15.93 | Physicians | 25.53 |
| School Teachers | 19.90 | All occupations | 22.83 |
| Lawyers | 20.22 | | |

Thus it appears that doctors not only furnish more victims than the members of any other profession, but their mortality rate is above the average of all trades and professions put together.

Claudius Ash & Sons, of London, England, the well-known manufacturers of dental materials, have, as may be seen by reference to their advertisement in this number, definitely determined to enter the American market, and have established a branch house at No. 30 East 14th Street, New York City, under the charge of Mr. C. A. Sykes, their genial representative who made so many friends in this country last year. It is unnecessary to speak concerning the reputation of this house. For many years it has occupied a leading position, honestly won and sturdily maintained. Gradually their business has extended until it has branch houses in Liverpool, Manchester, Paris, Berlin, Vienna, Hamburgh, Copenhagen and St. Petersburg. Heretofore the high American tariff upon goods of foreign manufacture has kept them out of this market, but they now find that they can pay the duties levied, and still compete with our own manufacturers in many things which are sold at much lower prices in England than in America. Their settling here will be an advantage to the profession, for their competition will be a wholesome one, and the excellence of their manufactures will give to American Dentists a yet wider variety of first-class material to select from.

---

According to the *Wiener Medicin Blaetter*, the inhalation of amyl nitrite is a reliable and rational antidote to cocaine intoxication. Its action is physiologically demonstrated by the fact that the action of cocaine on the vessels is contractile, and that of amyl nitrite dilative.

A case is reported, in which, for the purpose of painlessly extracting a tooth, six drops of a 20 per cent. solution of muriate of cocaine were injected between the gums and the alveoli. The amount of cocaine was therefore only 0.06 grams, but nevertheless, after the tooth had been removed, intoxication and insensibility set in. After several inhalations, from a cloth upon which were put three drops of amyl nitrite, the senses returned. Complete recovery resulted only, however, after repeating the inhalations for a number of times.

This treatment is more strongly recommended from the fact that the after-effects observed in poisoning by cocaine, such as loss of appetite, emesis, sleeplessness and weakness, are not apparent after the use of amyl nitrite.

—*Pharm. Rec.*

---

At the Recent Ophthalmological Congress in Paris, M. Paul Retard described a number of cases in which dental affections were evidently the cause of ocular disturbance, such as glaucoma, amaurosis, amblyopia, and cloudy vision. In asthenopia, without any apparent cause, the teeth should always be examined. M. Gayet mentioned a case in which disturbance was produced by a tooth fixed on a pivot; the symptoms appeared and disappeared according as the tooth was removed or replaced. M. Fieuxal had observed so many of these cases of correlation between ocular and dental affections that he had urged that a dental clinic be annexed to the Quinze Vingts Hospital for blind people. M. Suarez and M. Galezowski mentioned similar facts. M. Javal mentioned a series of cases of an inverse order, in which dental disturbance disappeared after operating for glaucoma.—*London Medical Record.*

Some of the Answers reported by the English Inspectors of Schools as having been given to special questions are exceedingly unique, and might perhaps be studied with profit by some of our dental physiologists. To the question "Describe the process of digestion," one of the "presented" pupils answered as follows: "Food is digested by the action of the lungs. Digestion is brought on by the lungs having something the matter with them. The food then passes through your windpipe to the pores, and passes off your body by evaporation, through a lot of little holes in the skin called capillaries. The food is nourished in the stomach. If you were to eat anything hard, you would not be able to digest it, and the consequence would be, you would have indigestion. The gall-bladder throws off juice from the food which passes through it. We call the kidneys the bread-basket because it is where all the bread goes to. They lay up concealed by the heart."

Another answer to the same question was this: "Food is digested when we put it in our mouths, our teeth chews it, and our tongue rolls it down into our body. We should not eat so much bone-making food as flesh-forming and warmth-giving foods, for if we did, we would have too many bones, and that would make us look funny."

On ventilation, one of the students informs us that "a room should be kept at ninety; in the winter by a fire, and in summer by a thermometer." Another says: "A thermometer is an instrument used to let out the heat when it is going to be cold."

---

"In the Claudian–Julian family, beginning with Julius Cæsar himself, and ending with Nero, we have an almost unbroken line of neuroses. Cæsar himself was epileptic, but probably the disease developed late in life, from exposure and excesses, and did not much affect his health.

"Augustus, his grand-nephew, had, it is believed, writers' cramp; Julia, his daughter, seems to have been little more than a nympho-maniac. She had an imbecile son. Tiberius was a man naturally heartless, cruel and licentious. In his later years he seems to have lost all moral sense, and illustrated the most shameless sensibility and cruelty. Caligula, reputed great-grandson of Augustus, was epileptic as a boy, badly formed and weak-minded as a man. He stuttered, was insomnious, and apparently had hallucinations. Claudius was also weak-minded, timid and credulous, with unsteady gait, weak knees, shaking head and dribbling lips —*New York Medical Record.*

---

The President of the French nation, Grevy, now nearly 80 years of age, lost his first tooth the other day. The Paris journals mention the fact as an event, and *The Voltaire* regards it as a matter of grave political importance. "Good teeth" says *The Voltaire*, "are essential to good digestion, and bad digestion has played a great role in the history of kings and rulers. It is lucky for us that President Grevy has such sound teeth. To that fact must be attributed his unchanging calm, his ataraxy, the perfect equilibrium of his physical and intellectual functions, which have made him the most prudent of statesmen and the most constitutional of presidents."

YOUNG DOCTORS, there is no help for it; you must practice on us of the laity before you can become really practical physicians. We will protect ourselves as long as we can. While the "old doctor" lives and is available we will, if we get sick, send for him. When we can do no better, we will send for you. Don't worry at this; it must be so; it is our only defense; you will have your revenge soon enough. The old doctor will die some day, or he will be too busy to come; something will befall us; the attack will be as sudden as severe; we must have your help or none. We may have laughed at you, and in our folly may have vowed that we would not send for you to treat a sick dog; but for all these follies of ours we will with desperate resolution send for you, trusting to a merciful Providence to help us through. (And we will trust Providence the more readily when we see even the young doctor as badly scared as his patient, at the bedside.) If we die you can explain it; if we get well we will sound your praises, even if we are slow in paying your fee.

If you kill a good many of us while really learning the practical part of your business, don't take it too much to heart, or throw your sign in the well, as Dr. Sims did after killing two or three patients.—*Atlanta M. and S. Journal.*

---

A REPORT on the newspapers of the world has recently been laid before the Imperial German Diet. It would appear that there exist 34,000 newspapers, the total issues of which, during the year, amount to 592,000,000. Of these, 19,000 papers appear in Europe, 12,000 in North America, 775 in Asia, and 609 in South America. 16,500 are in the English language, 7,800 in German, 3,850 in French, and about 100 in Spanish.

New York City is the great newspaper centre of the western hemisphere, as is indicated by the following table :

| | | | |
|---|---|---|---|
| Daily morning papers | 21 | Semi-monthly papers | 19 |
| Daily evening papers | 8 | Monthly papers | 168 |
| Semi-weekly papers | 7 | Bi-monthly publications | 4 |
| Weekly papers | 216 | Quarterly publications | 19 |
| Bi-weekly papers | 11 | | |
| | | Total | 473 |

---

DR. LOUIS ARNDT, of Jersey City Heights, when about to flask a piece for rubber work, fastens the center of a small, stout cord to the wax of the base on one side, carries it around the case and allows the ends to project from the flask. When the plaster has been poured and is partially set, the ends are carried back to the opposite side, drawing the cord through the plaster, thus cutting the investment into halves and allowing the two parts of the flask to be separated. Time is thus saved, and the use of shellac, oil, etc., becomes unnecessary.

---

A PARAGRAPH in the last number of this journal stated that the director of the United States Mint reported that the amount of gold consummed for dental supplies was $39,900, and silver $6,735. This is probably the amount that was withdrawn from the government depositories for this purpose. It could not cover the whole amount of the precious metals that are used by dentists.

THE "IDEAL BRUSHES," as attested by Dr. Barfield, to all who have used them, have proved "*Worth Their Weight in Gold.*"

In treatment of "Pyorrhœa Alveolaris" the extra-soft Prophylactic has been found of inestimable value, while the Dental Plate Brush is so essential that once used it becomes a necessity to both dentist and patient.

---

DR. F. A. GREENE, of Geneva, N. Y., avoids the dark lines in gum sections of rubber work by packing a piece of pink rubber in the joints. Cements, he says, dissolve out in time, leaving a space for the collection of fermenting food. By leaving the joints a little open, sufficiently so to draw a piece of paper through, there is no danger of cracking the sections.

---

IT IS OFTEN desirable to match shades in teeth at night, and this has heretofore been deemed impracticable. But if the teeth to be compared are viewed through the flame of a gas burner, or lamp, it will be found that all the varying shades become plainly perceptible, and in this way they can be selected with as much certainty as by daylight.

---

C. A. TIMME & CO., dealers in dental goods, Hoboken, N. J., desire us to announce that their senior partner has just returned from Europe, with new appliances and materials of great interest to the profession, which will, no doubt, be fully set forth in their advertisement hereafter.

---

DR. CARL SEILER says that some persons have a defect of smell analogous to color blindness. One person finds that violets smell like garlic, everything else smelling normally.

---

PHOSPHOR-NECROSIS of the jaw is becoming quite common in England, in consequence of popular self-prescriptions of phosphorus as a brain renovator. —*Med. and Surg. Reporter.*

---

PATIENT: "Well, doctor, what do you call it?" Doctor: "Progressive paralysis." Patient's wife: "Is it anything like progressive euchre, doctor?"

---

A CHINESE EDITION of "Gray's Anatomy," prepared by Dr. John Dudgeon, who has resided in China for over twenty years, has lately been published.

---

MORTALITY AMONG PHYSICIANS under the age of thirty is higher than that of any other profession during the same period of life.

---

DR. E. S. NILES, of Boston, has removed his office and residence from No. 136 to No. 241 Boylston Street, Copley Square.

---

OF THE fifty-five signers of the Declaration of Independence, five were physicians.

---

CHICAGO has eight medical journals, seven medical colleges, and six medical societies.

---

FOR PERSISTENT HICCOUGH give a pinch of snuff, and thus induce sneezing.

